# Asymmetric Power Relations in Gynaecological Consultations for Cervical Cancer Prevention: Biomedical and Gender Issues

**DOI:** 10.3390/ijerph18157850

**Published:** 2021-07-24

**Authors:** Carla Freijomil-Vázquez, Denise Gastaldo, Carmen Coronado, María-Jesús Movilla-Fernández

**Affiliations:** 1 Facultade de Enfermaría e Podoloxía, Universidade da Coruña, 15403 Ferrol, Spain; carmen.coronado@udc.es (C.C.); maria.jesus.movilla@udc.es (M.-J.M.-F.); 2 Laboratorio de Investigación Cualitativa en Ciencias da Saúde (LICcs), Grupo de Investigación Cardiovascular (GRINCAR), Universidade da Coruña, 15403 Ferrol, Spain; 3 Bloomberg Faculty of Nursing, University of Toronto, Toronto, ON M5T 1P8, Canada; denise.gastaldo@utoronto.ca; 4 Centre for Critical Qualitative Health Research (CQ), University of Toronto, Toronto, ON M5T 1P8, Canada

**Keywords:** cervical intraepithelial neoplasia, papillomavirus infections, physician–patient relations, professional–patient relations, emotions, gender identity, gender equity, feminism

## Abstract

A generic qualitative research, using a poststructuralist feminist perspective, was conducted in a Spanish gynaecology unit with the following aims: (a) to analyse how asymmetric power relations in relation to biomedical knowledge and gender shape the medical encounters between gynaecologists and women diagnosed with cervical intraepithelial neoplasia and (b) to explore the cognitive, moral, and emotional responses expressed by patients. A total of 21 women diagnosed with cervical intraepithelial neoplasia were recruited through purposive sampling. Semi-structured interviews were recorded and transcribed, and a thematic analysis was carried out. Two major themes were identified: (a) gendered relations in cervical intraepithelial neoplasia medical encounters are based on hidden, judgmental moral assumptions, making women feel irresponsible and blamed for contracting the human papillomavirus infection; (b) biomedical power is based on the positivist assumption of a single truth (scientific knowledge), creating asymmetric relations rendering women ignorant and infantilised. Women reacted vehemently during the interviews, revealing a nexus of cognitive, moral, and emotional reactions. In medical encounters for management of cervical intraepithelial neoplasia, patients feel they are being morally judged and given limited information, generating emotional distress. Healthcare professionals should question whether their practices are based on stereotypical gender assumptions which lead to power asymmetries during encounters.

## 1. Introduction

In Spain, as in many other countries, medical encounters in the context of cervical cancer prevention are permeated by dominant biomedical and gender assumptions that are rarely made explicit [1,2]. In this context, we believe gynaecological appointments should be conceived as a social encounter, where gender relations and biomedical knowledge intersect, creating specific conditions for understanding women’s health, gender roles, sexuality, and medical care. In this paper, we argue that the asymmetrical power relationships between doctors and female patients are perceived by the latter as moral judgment, rendering them irresponsible, guilty, ignorant, and infantilised.

### 1.1. Background

Human papillomavirus (HPV) infection is one of the most common sexually transmitted infections worldwide. Persistent infection with certain types of HPV causes cancers of the cervix, anus, vulva, vagina, penis, and oropharynx, as well as genital warts in men and women [3]. In 2020, an estimated 604,000 women were diagnosed with cervical cancer all over the world, and about 342,000 women died from the disease [4]. 

Health authorities suggest two main preventive strategies against cervical cancer: HPV vaccination and early detection of cervical precancerous lesions, known as cervical intraepithelial neoplasia (CIN), through cervical cancer screening programs [3]. In Spain, the National Health System (Sistema Nacional de Salud—SNS) offers free vaccination against HPV infection for girls aged 12 years [5] and free cervical cancer screening for women between 21 and 65 who engage in sexual activities [6]. However, immigrant women and women with lower socio-economic status are the groups who participate the least in screening programs [7]. For immigrant women, perceived social support and time living in Spain modulate to a large extent the differences in access to screening between immigrants and natives [8]. The screening is carried out by midwives in primary health care and consists of a pap smear every three years. When an abnormal cytological result is detected, patients are referred to specialised healthcare to confirm the diagnosis of CIN and plan medical surveillance (medical follow-up) or treatment based on the severity of the lesion (low-grade lesions (CIN 1) or high-grade lesions (CIN 2–3) [9,10].

In the scientific literature, several risk factors related to women’s behaviour have been identified as increasing the risk of contracting HPV infection and developing cervical cancer. These include smoking, not using condoms, prolonged use of oral contraceptives, multiparity, presence of other sexually transmitted infections (STIs), early age at first intercourse, and multiple sexual partners [11]. For this reason, during medical appointments, gynaecologists strongly advise women to quit smoking [12] and use condoms correctly and consistently [13] to reduce the risk of CIN progressing to cervical cancer. In addition, it is recommended that they receive the vaccine against HPV infection [14]. 

Internationally, scientific publications have focused on the biomedical aspects of CIN that support its prevention and clinical management. However, only a small group of researchers have explored healthcare experiences of women diagnosed with CIN. Some researchers have shown that, during CIN medical encounters, communication is limited and there is no adequate transmission of information from physicians to patients [15,16,17,18]. The hierarchical relationship inhibits women from expressing their doubts and concerns [16]. Women’s lack of knowledge about their condition reduces their faculty for self-care [19,20] and adherence to medical recommendations [19], as well as causing negative psychological feelings [21,22], such as anxiety [17,20] and fear of cancer [16,17]. Furthermore, the close relationship between CIN and sexuality, fidelity, promiscuity, and religious beliefs, makes women experience stigmatisation, self-blame, and shame [23], threatening the relationship with their partners [16,24,25] and making it difficult for them to request support from relatives and friends [25]. 

Our previous publications derived from this study have focused on exploring the experiences of women during medical surveillance for CIN [15,26]. The results showed that women who have a diagnosis of CIN experienced important healthcare informational challenges when accessing primary and specialised care, which had several repercussions on their wellbeing. The dearth of information provided and women’s limited knowledge about HPV infection and treatment made it difficult for them to understand and participate in decision-making about CIN prevention and treatment [15]. Additionally, we revealed how patients’ daily lives were affected by risk management in CIN medical surveillance [26]. Although doctors’ surveillance aimed to prevent the development of cervical cancer, women felt they were sick because they had to follow strict recommendations over an unspecified period of time and lived with the possibility of a life-threatening disease. Women with CIN reacted to the biomedical risk discourse in different ways: (a) complying with medical recommendations, which affected their sexuality and interfered with the possibility of motherhood, (b) increasing their personal risk management strategies beyond what is proposed by gynaecologists (e.g., women wanted to be under constant medical surveillance and requested treatment for their partners, avoided sexual intercourse, looked for other protective factors such as a healthy diet), and (c) resisting proposed risk management strategies (e.g., condom use, vaccination, quitting smoking) by questioning various elements of the prescriptions they received [26]. In this paper, we analyse healthcare users’ perceptions of their relationship with healthcare providers (gynaecologists) during CIN care and the cognitive, moral, and emotional responses these relationships created. 

### 1.2. Theoretical Framework

To further explore this topic, a poststructuralist feminist perspective is used, based on Foucault’s concept of biopower, focusing on the disciplinary power of biomedicine over sexuality [27]. We aim to analyse how power relations shape medical encounters between gynaecologists and patients diagnosed with CIN and the cognitive, moral, and emotional responses these relationships generate in patients.

For Foucault [27], biopower is a technology of power used by modern nation states to regulate subjects, through numerous and diverse non-coercive strategies (e.g., bio-politics, anatomo-politics), to achieve individual compliance and control of populations. Modern power, exercised through “discourses of truth” (e.g., biomedical discourse, gender discourse, sexuality discourse), becomes encoded into social practices, as well as human behaviour, as the human subject gradually acquiesces to subtle regulations and expectations of the social order [27]. According to Foucault [27], the promotion of biological life has become a political phenomenon in Western countries, supported by our current shared desire for healthy, long lives [28]. Population reproduction, health, and disease are fundamental to economic processes and are, therefore, subject to political control. From this perspective, biomedicine contributes to the exercise of biopower because it promotes norms of healthy behaviour (such as healthy sexual practices) that should be adopted by the entire population (for their own benefit), interfering with individual choice, and promoting and justifying discipline and surveillance for the achievement of good health [27,28].

Foucault locates the techniques of power (widely unintentionally exercised) in ‘banal’ institutions, such as the hospital and the clinic, especially in the interaction between doctors and patients during medical encounters (also referred to in this paper as consultations, appointments, visits) [29,30,31]. In understanding power relations as productive, Foucauldian theory states that medical dominance is necessary for practitioners to take control of the medical encounter and fulfil the expectations of both parties. Both the roles of the “doctor” and the “patient” are constituted by biomedical discourses and practices, which rest on the doctor being in the position of expert [32]. The doctor’s authority is based on the clinician’s biomedical or scientific knowledge associated with the ability to see the “hidden truths” of the body [29]. It is also derived from discourses of masculinity linked to the role of the doctor even when women are doctors [31]. In this context, detachment, reserve, responsibility for the patient’s well-being and an authoritarian stance must be maintained by the doctor. Conversely, the notion of patients being “empowered” to take control of the encounter makes little sense, for such a change in the relationship calls into question the *raison d’être* of the encounter [32]. Therefore, in the modern doctor–patient relationship, there is an expectation that “a good patient” will responsibly and voluntarily comply with medical instructions and expose the body to inspection by invasive, and many times embarrassing, tests and examinations so as to stay healthy and avoid disease [32]. Consequently, health promotion and disease prevention also moralise clients’ experiences, blaming those who have not been “good patients” and have irresponsibly ignored the recommendations [31,33,34].

Gender and sexuality discourses also shape the relationship between doctors and women during medical encounters. Foucault [27] argues that from the seventeenth century to the mid-twentieth century, Western societies became increasingly interested in sexualities that did not fit the marital union (such as homosexuality). Through the discourse of sexuality, rules have been established about sex and, consequently, certain sexual behaviours are socially accepted as normal while others are abnormal. Foucault argues that the “truth” about sexuality is examined through confession. He claims that the tradition of the Catholic confession became widespread, influencing relationships such as doctor–patient ones, in which the patients are the penitents and the doctors the authority figure to whom they must confess their (sexual) sins. The confession of sexuality is scientifically constituted as beneficial since it allows the identification of the “deviant” and “unhealthy” sexual behaviours [27]. Clinicians assert that they draw from rationality and science, rendering routine consultations free from moral judgment. However, sexual health (or gynaecology) involves “moral work”; doctors address sexual health problems in ways that differ substantially from non-STI-related health diagnoses [2].

Due to the reproductive power of women, the female body (and women´s sexuality) has been subjected to the scrutinising gaze of the health sciences much more than the male’s. Biomedicine has been built on masculine ideals for all human beings, pathologising female biology and legitimising inferiority and the need for the protection of women. Hence, there was a necessity to prescribe activities and behaviours for women (e.g., being a mother, being the main caregiver at home, as well as for relatives, being responsible for their own sexual health and the sexual health of their partners, and being sexually inhibited) [35,36]. Owing to the gender expectations associated with femininity, when a woman is diagnosed with CIN she must assume responsibility for having developed cervical lesions that may evolve to cancer, due to her “assumed” inappropriate sexual behaviour (in the case of CIN, being sexually active, having had multiple sexual partners, not having used condoms). This situation generates a high moral burden and individual guilt [1,26].

### 1.3. Aim

The aim of the study was twofold:

(a) To analyse how asymmetric power relations, in relation to biomedical knowledge and gender, shape the medical encounters between gynaecologists and women diagnosed with CIN and (b) to explore the cognitive, moral, and emotional responses expressed by patients.

## 2. Materials and Methods

### 2.1. Design

A generic qualitative methodology using a poststructuralist feminist perspective was developed. Caelli et al. define generic qualitative research as studies not guided by an explicit methodology in which the researchers create their own design choosing from different methods and theoretical perspectives [37]. In the health sciences, this is traditionally known as “qualitative research”. The advantage of using this approach is the guidance offered by Caelli et al. on how to make theoretical and methodological choices that are rigorous and consistent. Such an approach allows for sufficient flexibility in responding to the research objectives while focusing on quality during the whole research process [37]. Participants were recruited from a gynaecology unit in a public hospital of the Galician Health Care Service, located in the Northwest of Spain. The data generation method was semi-structured interviews, and a thematic analysis was carried out. 

### 2.2. Study Participants and Sampling Strategy

Participants were recruited from October to December 2015 through purposive sampling [38] following these inclusion criteria: to be a Spanish/Galician-speaking woman aged between 21 and 65 with a confirmed diagnosis of CIN of any degree. Women with a diagnosis of cervical cancer and physical or mental comorbidity interfering in the description of the phenomenon were excluded. 

The main researcher (C.F.V.), a nurse, joined the multidisciplinary gynaecology team during the consultations and made the study known to women with CIN after their appointments. In total, 31 women were initially interested in participating, so they were phoned to set an appointment where they were told C.F.V.’s academic background (BSc(N), MSc), the aims of the research, the arguments for carrying it out and what the participation consisted of. Finally, five women decided not to participate for personal reasons and another five did not attend the encounter without prior notice or justification. The final sample consisted of 21 participants. The sociodemographic characteristics of the sample are shown in Table 1.

### 2.3. Data Collection

Semi-structured interviews were conducted face-to face by C.F.V., using an interview guide created through literature review and the advice of three experts, two in qualitative methodology (M.J.M.F., C.C.) and one in CIN medical management. The interview guide was structured as follows: experience receiving the diagnosis, experience living with CIN and experience summary [26].

Most of the interviews lasted around 40 min. However, two of them lasted approximately 15 min. We perceived that these two women wanted to avoid sensitive matters. Other interviewees talked for more than an hour, providing in-depth explanations of their personal experiences.

The interviews were audio recorded and transcribed. Recordings were destroyed after verifying transcription accuracy. Field notes were written after each interview and integrated into the transcriptions. Data collection ended with the 21st interview when we reached information power [39].

### 2.4. Ethical Considerations

The study was approved by the Autonomous Committee of Research Ethics of Galicia (registration code: 2015/230) and by the hospital where it was conducted. An information sheet about the study was given to the participants and C.F.V. personally discussed potential participants’ doubts before they decided whether to volunteer. Written informed consent for participation in the study and publication of the results was obtained from all interviewees.

The interviews were held in a very accessible location, an office in the management area of the hospital, where no health professional could see interviewees because we were aware of the potential effect of the setting on interviewees confidence to speak openly about their experiences. This strategy also allowed for the anonymity of the participants and prevented interruptions by third parties (only the researcher and the interviewee were present during the meeting). Moreover, we perceived that the gender of the interviewer (a woman) made women feel comfortable during interviews when intimate aspects were discussed. The fact that C.F.V. had never worked in the gynaecological clinic of the hospital and had no previous relationship with the participants allowed the women to collaborate without considering that their participation in the research would negatively interfere with their healthcare. Due to the information needs expressed by the participants, C.F.V. answered questions posed by the women immediately after the interview and was available to talk at a later day, to quell their health concerns and created opportunities for participants to stop the interview at emotionally charged moments.

### 2.5. Data Analysis

A thematic analysis with inductive and deductive elements was performed inspired by the guidelines established by Braun and Clarke [40], Eakin [41,42,43], Eakin and Gladstone [44] and The Centre for Critical Qualitative Health Research [45], as well as our previous experience in qualitative data analysis. 

Throughout the process, analytical memos were developed and the ATLAS.ti software was used (version 7.5.10) to manage data analysis.

The data analysis was conducted in the following steps: (1) Familiarisation with the data: an initial listening of each interview was carried out, after which the recordings were transcribed verbatim; (2) Inductive analysis: each interview was read repeatedly to immerse the researchers in the experience of each participant. A code was assigned to each sentence/paragraph that represented an emerging idea with a code name and definition. After the coding process, the codes were classified into categories; (3) Deductive analysis: two emerging themes (the centrality of sexuality and biomedical knowledge in medical encounters) were identified leading to a poststructuralist feminist perspective deductive analysis of the data. Using this theory, we revised some of the initial codes and categories; (4) After the inductive and deductive phase of analysis we integrated categories into themes. Furthermore, comparisons were made between codes and categories belonging to the same theme, looking for similarities and differences in the analysis; (5) Defining and naming categories and themes according to the theoretical lens; (6) Producing the report: the final analysis was refined through the writing of this original article. The summary of the analysis is shown in Table 2.

### 2.6. Rigour

This report adheres to the Consolidated Criteria for Reporting Qualitative Research (COREQ) [46]. During the study, methodological and theoretical decisions were made following the guidelines established by Caelli et al. [37] to maintain the rigour and quality in generic qualitative studies. To ensure study trustworthiness [47], different verification strategies were utilised throughout the research. To achieve the credibility criterion, the verification of doubts was conducted during the interviews, since only one encounter took place per participant. Triangulation of sources during analysis was performed by three researchers (C.F.V., M.J.M.F., and D.G.). The final categories and themes were agreed by the whole research team. To reach confirmability, we used reflexivity and positionality strategies (e.g., discussing how our professional training influenced how we understood participants’ perspectives) and recorded all key methodological decisions throughout the study to be able to report them accurately. We also reflected on the relationship between the participants and the interviewer during data collection (see ethical considerations). Finally, the criterion of transferability and consistency were ensured by making a detailed description of the methodology used (study design, sample, sampling, data collection and data analysis) and of the study context.

## 3. Results 

Study participants ranged in age from 21 to 52 years. Overall, 15 of them were in gynaecological follow-up and 6 were in follow-up after conisation. The average time under medical follow-up was three years (ranging from one to ten years). Most were in a heterosexual relationship (19 participants), while two reported not having a partner. In total, 13 participants did not have children and eight were mothers (one or two children). 

Regarding power relations, gynaecological appointments should be conceived as a social encounter where gender relations and medical knowledge intersect, creating specific conditions for understanding women’s health, gender roles, sexuality, and medical care. Two major themes were identified: (a) gendered relations in CIN medical encounters are based on hidden and judgmental moral assumptions, making women feel irresponsible and guilty for contracting the HPV infection; (b) biomedical power is based on the positivist assumption of a single truth (scientific knowledge), creating asymmetric relations that render women ignorant and infantilised. 

### 3.1. Gendered Relations in CIN Medical Encounters Are Based on Hidden and Judgmental Moral Assumptions, Making Women Feel Irresponsible and Blamed for Contracting the HPV Infection

In most cases, CIN detection was performed by cervical cancer screening carried out by midwives in primary healthcare. Upon this abnormal finding, the patients were referred to specialised healthcare to confirm the result by colposcopy and biopsy and to plan the medical follow-up and treatment of CIN. The first medical encounter in specialised healthcare followed the usual order of medical consultations: anamnesis, communication of the results obtained in the cervical cancer screening and prescription of medical recommendations, and gynaecological examination and collection of samples from the cervix (Field notes: November 2015). When gynaecologists informed the women of their abnormal Pap smear (CIN), they explained that this condition was due to an STI, called HPV infection. 

During the anamnesis, the gynaecologists obtained information on the personal and family history of the women, allergies, menarche, duration of the menstrual cycle and menstruation, contraceptive methods used, history of pregnancies and abortions, smoking habits, number of sexual partners, results of previous Pap smears and vaccination against HPV infection (Field notes: November 2015). These questions were standardised during medical encounters and it was expected that women would answer them by confessing the truth.

Some of these questions asked about the sexual practices of the patients and addressed aspects socially linked to sexual promiscuity (e.g., number of sexual partners, non-use of condoms), irresponsible and inappropriate habits of a “good woman”. According to the participant´s accounts, they felt compelled to confess intimate matters of their sexual life, considering that their sexual behaviour (present or past) was being judged. Likewise, interviewee I-17 revealed that the gynaecologist had asked her questions during the medical interview (which were not included in the protocol of the anamnesis previously described) questioning how faithful she had been to her partner and the normality of their sexual relationships, making her feel offended and guilty. In the following quote, it is observed how the participant shares her experience with anger and irony.

*“She [the gynaecologist] told me it was something you caught [HPV infection]. At that time, I’d been with my partner for 11 years. The gynaecologist asked me if I’d had normal sexual relationships, if I’d been with the same partner for so long or [if I’d been with other people]… At that point I thought: ‘Yes, of course I’ve been with the same person. You have too, haven’t you? I suppose’. It’s like… really weird when someone says that, isn’t it? (…) I didn’t take it very well at that moment because I didn’t like the questions I was being asked, but I do understand they have to ask them, I suppose (…) How long I’d been with my partner, if sex was normal, if we led a normal life… I don’t know, what’s normal nowadays? I don’t know what’s normal. Something can be normal for me but not for you (…) I never felt guilty, but right then, being asked that question… I did”.*

I-17

The gynaecologists communicated the diagnosis of CIN to the women, explaining that it was caused by a sexually transmitted infection, HPV infection. The risk factors for developing CIN (e.g., having had multiple partners and unprotected sex) are described by the biomedical literature and taken for granted by society. The participants in our study compared these risk factors with their sexual practices (past or present) to understand why they had developed the disease. In accordance with this, interviewee I-2 assumed that she had to confess to the gynaecologist that she had performed risky sexual practices in the past, not using condoms with her previous partner, but was upset when she saw that the doctor reacted by blaming her for putting her sexual health at risk.

*“I felt a bit guilty about having that illness. [The gynaecologist] didn’t exactly say ‘it’s your fault you’ve got it’. No, she seemed to suggest that she couldn’t believe I hadn’t been advised when I was younger about taking precautions [using a condom]. But of course, I was in a steady relationship and was taking the pill [contraceptive], I trusted him [her partner] (…) I was upset when I left [the consulting room], I wouldn’t say she treated me badly, she didn’t hit me or anything, she didn’t even insult me, but if someone you’re supposed to open up to treats you like that and blames you for not taking the precautions you’re supposed to have taken…”. *

I-2

On the contrary, some women were thinking about the reasons why they had contracted HPV infection, since they had acted responsibly to maintain their sexual health by not having had multiple sexual partners, having used condoms during sexual intercourse, or having previously been vaccinated against HPV infection.

*“I’d been vaccinated, been steady with a partner for seven years, it was almost like being a girl who’d never had sex before. It could have taken effect [the vaccine], you know? (…) I wasn’t one of those risk factor cases (…) a smoker, taking contraceptives [oral], having sex with different partners (…) So I thought: ‘damn, why me?’”.*

I-10

Some gynaecologists were aware of the impact caused by the diagnosis of CIN in women and tried to avoid judging their situation using biomedical discourse, reporting on the prevalence data of HPV infection. According to the descriptions of some participants, knowing these data helped to reduce the feeling of blame generated by “having had too many sexual partners” and reassured them to know that many other women were in the same situation.

*“It’s reassuring to think ‘I didn’t catch this because I’d slept with all the guys’ (…) Yeah, that calmed me down a lot. So often I’ve heard [gynaecologists tell us] about the number of women who’ve got it and that it’s common among women of 30”.*

I-18

Participants in our study expressed a preference for the healthcare offered by male gynaecologists, describing that they had been “gentler and politer” with them during medical encounters than female gynaecologists. Patients expected to be understood and not be judged by female gynaecologists, since they considered that, by sharing the same sex, they shared patriarchal experiences of discrimination and they might have faced this same health problem. However, most of the participants showed their perplexity at the lack of empathy of the female doctors, which made them feel misunderstood, helpless, and vulnerable.

*“With some [gynaecologists] it went really well, but with others it unfortunately didn’t. I hate to say so. In my experience the two men gynaecologists were like more sensitive, more tactful when telling me [the diagnosis] (…) Some of the women gynaecologists not as much (…) Sometimes I thought: ‘blimey, well I can’t believe it couldn’t happen to you [to the female gynaecologist]’ (…) Take my first appointment for example, [mentions male gynaecologist’s name] told me: ‘Well, my daughter also had it and she was a CIN 1 and she reacted the same way [upset and crying]’ The way you say it… matters, you know?”. *

I-10

*“She [the gynaecologist] can’t tell me it’s my fault because, maybe, she does the same thing with her husband [not use a condom] (…) Loads of things cross your mind and you think: ‘Well, I might as well shut up because it’s not her fault, the poor thing, I’m not going to mix her up in this.’ But sometimes you think: ‘shit! I do it and you do it too!’ She was very distant and cold. On top of that, I was alone, being given some bad news… without anyone to answer back [to defend her from the gynaecologist’s accusations]…”. *

I-2

Complying with medical recommendations to prevent CIN from progressing to cancer was based on the assumption that women and their partners should change their sexual habits in a consensual manner (e.g., consistent use of a condom). However, the absence of women’s partners during medical encounters showed that the responsibility for having had risky sexual relations and caring for the sexual health of both members of the couple fell exclusively on women, a situation that women questioned.

*“I think they [their male partners] should also participate [comply with medical recommendations to prevent CIN becoming cancer] (…) I think they should say [gynaecologists to women]: ‘look, come along with your partner’. Even tell them [the partners]: ‘you’ve got to use a condom, if not you’ll infect your partner’”. *

I-15

This same idea was ironically described by interviewee I-14, alluding to the non-vaccination of men, when they also acquire and transmit HPV infection.

*“Forget him [her partner]! He’s worse than useless. He’s got no idea [of what HPV infection is], and his name didn’t come up anywhere [no gynaecologist named him] (…) If the laboratories knew anything they’d take measures wherever it was transmitted [the virus], wouldn’t they? Because we catch it, put up with it and bear it, but they [the men] are the ones spreading it. Instead of doing something so that men can’t have it, so they won’t pass it on to me… well, they make a wonderful and expensive vaccine for me, so I’m protected”. *

I-14

### 3.2. Biomedical Power Is Based on the Positivist Assumption of a Single Truth (Scientific Knowledge), Creating Asymmetric Relations That Render Women Ignorant and Infantilised

The asymmetric power relationships established between gynaecologists and women during medical encounters positioned gynaecologists as experts in the pathology and its clinical management, while women were seen as inexperienced subjects, unaware of their own health, body, and self-care. This situation generated the expectation that women should comply with medical recommendations (smoking cessation, use of condoms, vaccination against HPV infection) and to expose their bodies to prevent their cervical lesions (CIN) from evolving into cancer, understanding surveillance and medical monitoring as beneficial, without considering the negative effects this could have for them. 

Women responded in different ways to these asymmetric power relations. Some women assumed that both the paternalistic attitude of gynaecologists and exposing their bodies to gynaecological examination were beneficial in keeping them healthy. However, they were also upset with the treatment received, emphasising the need to receive symmetrical, close, empathetic, individualised, and non-judgmental treatment during gynaecological consultations, since issues related to their deeper intimacy were addressed in a manner that made them uncomfortable. The quotation from interviewee I-2 shows this idea, in addition to reflecting her bewilderment at receiving the news of an unexpected and serious condition (CIN).

*“Just like the doctor told me the other day: ‘you’re 23, young, don’t worry but you’ve got to…’ [giving her medical recommendations she should follow] like telling me off, but nicely, tactfully, realising that I’ve got an important illness, which I didn’t expect. I don’t want her [the gynaecologist] to know about my life, or to ask questions about it, but I’d like her to… as she doesn’t know about my life, be a little more tactful. Because I don’t know about hers and I’m not going to get into her life (…) She was very cold towards me… I’ve got to spread my legs in front of her, you know?! She should be a little more sensitive and human. I know she’s got lots of patients, she’s got to attend many people and they’ve all got things she’s got to deal with… but I haven’t told her a thing about my life. She gave me the news! So, at least, considering I’m stuck and all I do is cry, well… girl, be a bit tactful! Just a bit”. *

I-2

However, other women were reluctant to accept the paternalistic attitude of gynaecologists. According to the accounts of some participants, they felt that gynaecologists infantilised them by constantly reminding them “what to do” and invalidating their knowledge. Most of the participants did not show their anger to the gynaecologists. However, interviewee I-13 recounted a situation of confrontation with the doctor due to this situation.

*“As soon as I walked in [to the consulting room] I felt like a little girl. Like I was being told off and I’m 35 years old, I don’t want anyone telling me off because I know what I know, I’m a grown up, I’m not 15 so I don’t want them [the gynaecologists] to say: ‘hey, remember to use a condom’. ‘Look, madam [she said to the gynaecologist], I’m a grown up, don’t treat me like a little girl, I’m older than I look. I know full well what I can and can’t do (…) It was almost like standing up to her. I said: ‘look here…’”.*

I-13

The technical language used by gynaecologists maintained the asymmetrical relationship between doctors and patients and dehumanised medical care. The participants stated that they did not understand the language used by gynaecologists and that they limited themselves to providing them the information they considered relevant, without giving them the opportunity to express their doubts, concerns or actively participate in conversations. This aroused negative feelings (such as fear, lack of trust) and suggested they had to blindly trust medical recommendations without understanding, on many occasions, the reasons for carrying them out.

*“[The gynaecologist] used words I didn’t understand (…) As if it weren’t person to person. Here’s a doctor who’s saying things you don’t understand and what that does is frighten you”.*

I-7

*“The moment everything’s being explained [by the gynaecologist], it’s all cold, hard data (…) I tried interrupting to ask a question and she said [not to interrupt her]. If I do interrupt it’s because I don’t understand or that I’ve got a doubt. Later, I’ll probably forget and leave without having asked (…) That passing on of information seemed rather curt. [The gynaecologist] doesn’t let you take part in that transfer of information. It’s like I had to listen to everything, at that moment I wasn’t 100% calm, I was a bit nervous and… I’d like to take part more, be able to interrupt and ask when I’ve got any questions”.*

I-3

The gynaecological exams were perceived by a few participants as an assault and an invasion of their privacy. However, they considered that the gynaecologists were not aware of this. This could be because doctors focus only on the benefits that this medical procedure has for women.

*“I think that Health Professionals don’t understand how aggressive [gynaecological examination] can sometimes be (…) They are very intimate issues, they’re very personal. They lay you on a bed in a room full of lights and a spotlight and… I know doctors aren’t thinking: ‘I’m looking at this woman’s vagina’. But really, they don’t put a face to the body. When I leave I sometimes say to myself: ‘damn, they’ve been putting things [in my vagina]’. You feel attacked, but gynaecologists don’t really understand. Just a smear is OK, but a biopsy, colposcopy and the doctors all there looking on… is quite violent”.*

I-18

With the expression of the previous quote “they don’t put a face to the body”, participant I-18 showed the dehumanisation of gynaecological care, reflecting that gynaecologists focused on the pathology of the genitals and not on the woman in front of them. Furthermore, according to the participants’ descriptions, the pain caused by gynaecological procedures (especially cervical biopsy) and the lack of information about them, made the gynaecological examination uncomfortable and frightening.

*“She [the gynaecologist doing the biopsy] said: ‘It’s not going to hurt’ (…) I was really uncomfortable and to make matters worse [she said]: ‘don’t move’, ‘but I can’t stop moving because you’re hurting me’ [she replied to the gynaecologist]. ‘I’ve got to take it, there’s just a bit left’ [the gynaecologist says] And I said: ‘fuck… a bit… a bit of what?’. It’s not pleasant and what’s more, it’s something I’ve never done before. I thought: ‘Jeez, that’s hurts loads!’ On top of it all I was nervous about everything she’d said… That was a very bad day, I think it was one of the worst days in my life”. *

I-2

Assumptions about gender as well as biomedical knowledge and practices shape gynaecological appointments for CIN follow-up. Women perceived these encounters as moral judgment, and the asymmetric relationship established with gynaecologists made them feel ignorant and incompetent for self-care. They reacted to these effects in a visceral manner, showing anger and irony, in addition to feeling offended, upset, misunderstood, helpless, bewildered, scared, and attacked.

## 4. Discussion

This study offers an analysis of how asymmetric power relations shape medical encounters between gynaecologists and women diagnosed with CIN as well as their cognitive, moral, and emotional responses to this experience. Gendered relations and biomedical knowledge shape not only the practices of physicians but also of other healthcare professionals, such as nurses and midwives, who in other countries would perform some of the clinical care described here as performed by gynaecologists.

As mentioned above, we classified our main findings into two themes: (a) gendered relations in CIN medical encounters are based on hidden, judgmental moral assumptions, making women feel irresponsible and blamed for contracting the HPV infection; (b) biomedical power is based on the positivist assumption of a single truth (scientific knowledge), creating asymmetric knowledge relations that render women ignorant and infantilised. They reacted to these encounters vehemently during the interviews, revealing a nexus of cognitive, moral, and emotional reactions that are difficult to describe separately. *Grosso modo*, from a cognitive perspective, women could not understand some information, challenged the prescriptions received or could not understand the rationale for recommendations [15,26]. Morally, they described being blamed for having acquired HPV infection, as well as irresponsible and unable to take care of themselves. From an emotional perspective, they showed anger and irony, in addition to feeling offended, upset, misunderstood, helpless, bewildered, scared, and attacked. From a feminist poststructuralist view, it is important to explore the effects of power relations, and in the case of the study participants, the diagnostic and follow up of an STI produced a cognitive-moral-emotional burden that we consider unnecessary suffering, but typical of a patriarchal society where medicalisation is seldom questioned if patients are cured [48,49]. 

Gynaecological history and examination protocols in Western countries include asking questions about women’s sexual practices (e.g., frequency of sexual activity, number of sexual partners, use of contraceptives, use of condoms) to identify risk factors, correct unhealthy habits through health education and determine if it is necessary to perform additional examinations or tests [50,51,52]. According to our participants, most gynaecologists applied these guidelines during anamnesis, expecting women to disclose information about intimate relationships, without considering the negative impact of these questions. Although doctors seemed to have treated the information as neutral, according to Foucault [27], these confessional practices have occurred within the doctor–patient power relationship for centuries now as a form of power exercise. The authority (in this case, the gynaecologist) expects confession and intervenes by judging, forgiving, or consoling. Some participants were confused or angry with such an “examination” as they did not meet the characteristics of the risk profile described by biomedical literature, which reveals that this risk profile is not applicable to all women who are diagnosed with CIN.

The results of our study coincide with those obtained by other authors in that the asymmetric doctor–patient relationship causes women to be inhibited when expressing their information needs and concerns [16] and that the technical language used by doctors makes communication difficult between both parties [15,53]. According to the descriptions of the study participants, gynaecologists adopted a paternalistic attitude and infantilised them, constantly telling them how they should act to prevent CIN from evolving into cancer, underestimating their ability to make adequate decisions about their self-care. As described in our previous publication [26], this paternalistic attitude is the consequence of the expectation of women’s compliance with medical recommendations, even though, in some cases, they do not understand the reasons for carrying them out. Such compliance (e.g., use of condoms) implies effects on the personal and social lives of women diagnosed with CIN (e.g., conflicts in the relationship with their partner, changes in their sexuality, delay in pregnancy plans) that are not considered during medical encounters. These effects cause women to resist gynaecological instructions [26]. 

Women participate in surveillance medicine as part of being a ‘responsible’ (female) citizen; as a woman who ‘takes care’ of her health. Participation in cervical screening is presented in contemporary public health literature as a normal part of womanhood [54]. However, even though these gynaecological examinations are standardised and considered beneficial by gynaecologists and patients, our study participants described that they felt uncomfortable and embarrassed during gynaecological examinations. Similar results were obtained in other studies where it was shown that women suffered anxiety, distress, and pain during gynaecological examinations [55,56]. 

Gendered relations in CIN medical encounters make women perceive that they are being subjected to a moral judgment. Despite the shared belief that biomedicine is based on scientific principles of objectivity and the ethical principle of altruism, moral values permeate the medical encounter [32]. As described by Cook [2], gynaecologists routinely act as moral educators, although most of the time unintentionally. By working from an evidence-based model, physicians operationalised in the clinic what is stated by the biomedical literature—that the risk factors for HPV infection, CIN, and cervical cancer are related to sexual behaviour (e.g., not using condoms, having multiple sexual partners) [11]. As observed in our study, women with CIN felt judged when asked about their sex lives, due to the social belief that certain behaviours are not typical of a “good woman” and are linked to promiscuity [23]. As Armstrong [1] states, sexually acquired conditions are morally charged and potentially carry implications of individual responsibility and guilt. 

The biomedical literature establishes that the greater the number of sexual partners, the greater the probability of developing CIN [57]. However, we consider that “having had multiple sexual partners” is a very subjective risk factor that has no predictive value. Multiple partners can be understood as having had more than one or having had incalculable sexual partners, and this ambiguity does not help in CIN diagnosis, nor prevention of cancer. Rather, it generates stigma, since women must assess the extent to which they believe they have been promiscuous and irresponsible based on the number of partners they have had, when they could have acquired HPV infection with their first sexual partner. As healthcare professionals, we believe that any woman who has had sexual intercourse, regardless of the number of sexual partners, can contract HPV infection and thus develop CIN in the future. This idea needs to be conveyed during consultations and it is time to question the need for information on the number of sexual partners. This moral burden is greater due to the patriarchal expectation that women should take care of their sexual health and be sexually inhibited [35,36].

Moreover, the responsibility for care was attributed to women by default as gynaecologists do not include their (male) partners during consultations. This situation surprised study participants, since following the medical recommendations implied making changes that had to be negotiated and potentially acquiesced by their partners. As suggested by Thompson [36], gender and biomedical discourses discipline women as responsible for sexual and reproductive health while relinquishing male responsibility. In addition, individualistic imperatives disregard the role men have in women’s sexual health.

Feeling irresponsible, blamed, ignorant, or infantilised over a series of medical appointments created emotional responses, mainly characterised by anger, which varied from feeling upset to feeling attacked. The participants’ reactions point to the multiple dimensions of the clinical encounter (social, political, medical), where questions about sexual practices are framed within dominant discourses about women’s sexuality in a patriarchal society and no neutral way of talking about one’s own health and the possibility of developing cancer exists [1,36,58].

As Ahmed [59] proposes, emotions are not only related to the impressions left by others but are also created by social norms. Emotions are brought about by gender, sexuality and biomedical discourses, and are also the cause of resistance to asymmetric power relationships created during medical encounters. As a consequence, discontentment with relationships often makes individuals challenge social norms that frame such encounters. Interestingly, most participants did not disclose how they felt about the consultation, with only one woman confronting the gynaecologist showing her discomfort at being infantilised. We assume that access to life saving treatment delivered by doctors prevented women from expressing themselves freely. However, many participants told us they volunteered to participate in this study because they wanted CIN care to be changed. 

Based on participants’ experiences, we believe some gynaecologists are aware of the negative effects of the CIN follow-up process as they used epidemiological data (specifically, prevalence data) to normalise the diagnosis of CIN, counteract stigma, and reduce the moral burden of having acquired an STI. Resembling the results obtained in other studies [2,58], these strategies used scientific “truths” to reduce moral judgment.

It should be noted that some women in our study expressed their preference for the care provided by male gynaecologists. This finding is supported by the results obtained by Roter et al. [60], who describes that male gynaecologists show higher levels of emotionally-focused conversation than female gynaecologists. According to the descriptions of our participants, we believe that the preference of some of them for male gynaecologists was due to their expectation of greater empathy from female gynaecologists. Not feeling understood by them, they felt even more judged. Although Lupton [31] reminds us that the power relationship between doctors and patients is intended to place the doctor in the position of an expert with authority (e.g., medicine’s high status as a male profession) and maintain the reason why the medical encounter exists, we believe our participants had sexist expectations for female gynaecologists (supposing they would be “naturally” warmer and kinder because they were women).

## 5. Conclusions

The theoretical approach used has allowed us to identify how the dominant discourses (biomedical and gender) shape the power relations between gynaecologists and women diagnosed with CIN, causing several cognitive-moral-emotional responses in patients. CIN medical encounters are perceived as offering limited information, as a form of moral judgment, and creating emotional distress. Our study results can be applied by health professionals working with women’s sexual and reproductive health, such as gynaecologists, family doctors, nurses, and midwives.

To offer better care, healthcare professionals should think about the clinic within society, understanding that biological issues are also political (power imbued) issues [27]. From a poststructuralist perspective, the current forms of power exercise in CIN follow-up and treatment are based on the dominant assumption of the neutrality of medical practice that have “adverse”, disciplinary effects for healthcare users. However, our theoretical framework also raises some difficulties when implementing supposedly improved healthcare practices [28] because reducing power asymmetries could be conceived as a more efficient way to exercise power and reduce resistance [28,61]. In this paper, we have prioritised the emic perspective of participants who would like to see changes in the way women are treated in CIN care, even though we understand these subtler forms of biopower and biopedagogy will require further analysis of power relations in health care [28,61].

Considering our participants’ experiences, we propose healthcare providers should expand the way they think about healthcare delivery; taking into account women’s non-biomedical rationalities, self-care skills, and the need to respect their decisions could potentially address the individual and social dimensions of sexual diseases and their implications for women. Questions regarding women’s sexual practices should be avoided in gynaecologic history protocols of CIN. Obtaining this information does not change the current illness situation of the patient, yet has a negative impact on their self-perception as women. Healthcare should be offered to both women and their partners (who may be male, female, non-gender compliant, cis, or transgender) to reduce the negative personal impact of being solely responsible for the management of CIN. 

This study provides an original contribution to the understanding of power relations between women and gynaecologists during the prevention of cervical cancer in Spain and to the field of feminist health analysis in the context of cervical cancer internationally. We have obtained rich data that have allowed us to explain multiple aspects of the experience of patients in the Spanish National Health System, pointing out the need to include a gender perspective to improve healthcare (emic perspective). Being this a novel and exploratory study, it has some limitations that researchers in the field should consider. To obtain more specific results in future research, we recommend investigators should design studies using a purposive sample to characterise the experiences of groups such as those who experience inequities in access and utilisation of health services, patients of different ages, educational levels, sexual orientations, race/ethnicity, and citizen-migratory status to achieve more nuanced understandings regarding power relations and the ability to negotiate information and emotions in the clinical encounter. In addition, more research is needed to explore the relationship between women with CIN diagnosis and different health professionals who assist them at different levels of healthcare (gynaecologists, family physicians, midwives, and nurses), as well as interprofessional collaboration for consistency of meaningful care for women. 

## Figures and Tables

**Table 1:** Sociodemographic factors.

Participant	Age	Academic Level	Marital Status	No. of Children	Type of Diagnostic	Year of Diagnostic
I-1	33	Graduate	Single (with partner)	0	CIN 1	2013
I-2	23	Elementary School	Single (with partner)	0	CIN 2	2015
I-3	29	Higher Professional Training	Single (without partner)	0	CIN 1	2008
I-4	46	Graduate	Married	2	CIN 1	2015
I-5	25	Graduate	Single (with partner)	0	CIN 3	2015
I-6	33	Bachelor’s degree	Single (with partner)	0	CIN 3	2013
I-7	21	Medium Professional Training	Single (with partner)	0	CIN 1	2012
I-8	26	Graduate	Single (with partner)	0	CIN 1	2014
I-9	45	Elementary School	Married	2	CIN 1	2014
I-10	42	Graduate	Married	1	CIN 3	2010
I-11	39	Higher Professional Training	Separated (with partner)	1	CIN 2	2012
I-12	27	Graduate	Single (with partner)	0	CIN 2	2014
I-13	35	Higher Professional Training	Married	1	CIN 1	2015
I-14	52	Graduate	Married	1	CIN 1	2010
I-15	25	Higher Professional Training	Single (without partner)	0	CIN 1	2012
I-16	37	Medium Professional Training	Single (with partner)	0	CIN 1	2007
I-17	29	Graduate	Married	0	CIN 1	2013
I-18	26	Graduate	Married	0	CIN 1	2010
I-19	48	Medium Professional Training	Divorced (with partner)	1	CIN 1	2015
I-20	44	Bachelor’s degree	Married	2	CIN 3	2005
I-21	34	Higher Professional Training	Single (with partner)	0	CIN 1	2014

Note: I stands for “Interviewed”, CIN 1 stands for “Low-grade cervical intraepithelial neoplasia”, CIN 2/CIN 3 stands for “High-grade cervical intraepithelial neoplasia”.

**Table 2:** Summary of analysis.

**Theme** **1:** **Gendered Relations in CIN Medical Encounters Are Based On Hidden, Judgmental Moral Assumptions, Making Women Feel Irresponsible and Blamed for Contracting the HPV Infection.**	
***Categories:***	***Codes:***
Events that make women feel judged and blamed.	Intimate questions during the anamnesis. Performing risky sexual practices. Not performing risky sexual practices. Assuming responsibility of the couple’s risky sexual practices.
Events that reduce women´s moral burden.	Knowledge of epidemiological data of the HPV infection.
**Theme** **2:** **Biomedical Power is Based on the Positivist Assumption of a Single Truth (Scientific Knowledge), Creating Asymmetric Relations that Render Women Ignorant and Infantilised.**	
***Categories:***	***Codes:***
Women’s experiences during the prescription of medical recommendations.	Feeling infantilised due to the paternalistic attitude of healthcare providers. Acceptance of the paternalistic attitude of health providers. Healthcare providers´ lack of empathy. Language not understandable. No opportunity to express doubts and concerns. No opportunity to actively participate during consultations. Preference for male gynaecologists.
Women’s experiences during gynaecological examinations.	Duty to expose their bodies to be explored. Discomfort. Pain. Aggression. Dehumanisation. Misinformation.

Note: CIN stands for “Cervical Intraepithelial Neoplasia”, HPV stands for “Human Papillomavirus”.

## Data Availability

There is an ethical and legal restriction on sharing our data. Data contain potentially identifying and sensitive patient information. According to the Autonomous Committee of Research Ethics of Galicia (Spain) (email: ceic@sergas.es) only the authors of this study can have access to the data.
